# Trends in malaria admissions at the Mbakong Health Centre of the North West Region of Cameroon: a retrospective study

**DOI:** 10.1186/1475-2875-13-328

**Published:** 2014-08-22

**Authors:** Ignatius C Ndong, Mari van Reenen, Daniel A Boakye, Wilfred F Mbacham, Anne F Grobler

**Affiliations:** DST/NWU Preclinical Drug Development Platform, North-West University, Potchefstroom Campus, Vanderbijlpark, South Africa; Department of Health Economic Policy and Management, School of Health and Medical Sciences, Catholic University of Cameroon, Bamenda, Cameroon; Laboratory of Public Health Research Biotechnologies, Nkolbisson, University of Yaoundé I, Yaoundé, Cameroon; Noguchi Memorial Institute of Medical Research, University of Ghana Legon Accra, Accra, Ghana; Statistical Consultation Services, North-West University, Potchefstroom Campus, Vanderbijlpark, South Africa

**Keywords:** Malaria, Prevalence, Mbakong, Cameroon, Trends, Treatment, Policy

## Abstract

**Background:**

Malaria is the leading cause of death worldwide. It is urgent to assess the impact of interventions and scaled-up control efforts. Despite reported reduction in malaria prevalence in Africa, the trends in Cameroon are not yet fully understood. The aim of this study was to investigate the trends in malaria admissions among febrile patients seeking treatment over a seven-year period (2006–2012) in an endemic area in Cameroon, hypothesizing a declining trend. This period followed changes in malaria treatment policy. The objectives were to identify possible trends in malaria admissions and to evaluate the impact of changes to treatment guidelines on the prevalence.

**Methods:**

Data was collected through consultation and perusal of laboratory and prescription registers of the Mbakong Health Centre. Data analysis was conducted using SPSS and SAS Statistics.

**Results:**

Analysis revealed that 4,230 febrile patients were received from 2006–2012. Of these febrile cases, 29.30% were confirmed positive. Between 2006 and 2012 confirmed malaria positive cases of those tested fluctuated, dropping from 53.21% in 2006 to 17.20% in 2008; then rising to 35.00% in 2011 and, finally, dropping to 18.2% of those tested in 2012. The prevalence in females and males across all age groups were similar: a slightly higher risk of males to have malaria (OR = 1.08, 95% CI 0.94-1.25) were not practically significant. Of those tested, the 5 to < 15 years and the 1 to < 5 years age groups were the hardest hit by malaria in the area. A practically visible and significant association was observed between the age and gender with regards to the number of malaria positive results (Pearson ×^2^ = 153.675, p < 0.00001, Cramer’s V = 0.352). Malaria prevalence exhibited a fluctuating yet declining trend, as observed over the 28 quarters between January, 2006 and December, 2012.

**Conclusions:**

The changes to the treatment guidelines appear to result in a declining trend as was observed between 2006 and 2008. However, malaria admissions fluctuated between 2008 and 2012. There is, therefore, a need to step up control efforts of especially the vulnerable groups, such as the very young.

**Electronic supplementary material:**

The online version of this article (doi:10.1186/1475-2875-13-328) contains supplementary material, which is available to authorized users.

## Background

Malaria is a complex disease that varies widely in epidemiology and clinical manifestations and remains the leading cause of death in the world [1, 2]. The 207 million malaria cases reported globally in 2012 resulted in an estimated 627,000 deaths with 80% of the cases in Africa [3]. In 2010, malaria was responsible for 24% of child deaths in Africa [4]. The world malaria report of 2013 further revealed that between 2000 and 2012 the malaria mortality among Africa children dropped by 54%. Malaria is endemic in Cameroon with a prevalence rate of 29% [5]. It is the major cause of morbidity and mortality among the most vulnerable groups: children under five (18%), pregnant women (5%), people living with HIV/AIDS (5.5%), and the poor (40%), the last of which constitutes two thirds of the total population estimated at 19 million [6]. Close to one million clinical cases of malaria occur annually, accounting for more than 50% morbidity among children under age five, 40 to 45% of medical consultations, and 30 to 47% of hospitalizations [7]. In addition, it is responsible for 49% of prenatal consultations and 59% of hospitalizations during pregnancy leading to abortions, premature labour and deliveries and low birth weight (LBW), thereby resulting in infant and maternal mortality [8, 9].

The epidemiologic profile of malaria in Cameroon appears to be aligned along three geographical and climatic regions: i) an endemic and perennial transmission zone covering the southern equatorial forest, coastal and western plateau with 7–12 months of rainfall, ii) an endemic and seasonal transmission zone in the Adamawa plateau and savannah forest with 4–6 months of rainfall and iii) an epidemic and strongly seasonal zone covering the Sudano-sahelian region with seasonal transmission of 1–3 months [9, 10].

The main methods for malaria diagnosis have been by clinical diagnosis and microscopy until recently, when rapid diagnostic test (RDTs) were introduced and the Cameroon government rolled out RDTs in 2012 to improve health facility diagnoses and home based management of malaria. Microscopy as a testing service is widely available (at 91% of public and 100% of private facilities), but reportedly underused, with less than half of febrile patients tested for malaria during their consultation in some countries in sub-Sahara Africa [5, 11].

Over the last decade, the Cameroon government has taken a series of steps to reduce the burden of malaria. Owing to the development of resistance to various anti-malarial drugs, the government adopted the WHO recommendations of 2001 and changed the treatment policy from chloroquine to artesunate-amodiaquine (ASAQ), as the first-line treatment for uncomplicated malaria in 2004 and endorsed artemether-lumefantrine (AL) as an alternative artemisinin combination therapy (ACT) in 2006, following alleged side-effects linked to ASAQ. Under this new policy quinine and artemether injections are reserved for cases of severe malaria, and sulphadoxine-pyrimethamine for intermittent preventive treatment during pregnancy [5, 12–14].

Although the impact of interventions on malaria prevalence may differ from country to country [15], there is mounting evidence across Africa that the increasing level of malaria interventions have led to a reduction in malaria prevalence from 37% in the years 1985–1999 to 17% in 2000–2007 and a consequent decline in morbidity and mortality [16, 17]. This has been brought about through combining ACT and vector control using long-lasting insecticidal nets (LLIN) [4]. In Rwanda, in-patient and outpatient malaria cases in the under five age group decreased by 55% and 67% respectively and in Ethiopia by 73% and 62% following a nationwide distribution of LLIN and artemisinin-based combination therapy (ACT). In Cameroon there has been an overall reduction from 41% in 2008, through 36% in 2010 to 31% in 2011 while malaria related deaths reduced from 29% in 2009 to 19% in 2011. In this country, free malaria treatment was started for all children under five years of age in 2011. In addition, community based management of malaria using community relay agents was started and in 2012 more than eight million free mosquitoes nets were distributed to families nationwide [18–21]. The latter was a scale up of the already existing programme of free mosquito net distribution to pregnant women and under five children who visit antenatal clinics [22–24]. Maternal mortality due to malaria also decreased from 5% in 2010 to 1% in 2011 [21]. This could lead to a change in the transmission prolife of different zones [25]. Thus, the WHO in 2010 recommended parasitological confirmation of all malaria cases before treatment for patients five years and above where testing services are available [26, 27]. This was geared towards reducing the unnecessary exposure to anti-malarials.

If malaria prevalence is decreasing as a result of interventions, it is expected that the number of malaria positive cases will decline in an area where such interventions are taking place. Further investigations should also be conducted to identify the proper course of treatment for malaria negative febrile cases [28, 29]. In addition, the reduction in prevalence should be evident and significant across different age groups, gender and over time. The data analysed in this study spanned a seven-year period (2006–2012). Malaria resurgence is known to follow a five- to six-year epidemic cycle [30, 31], which is most probably an inherent characteristic of the disease itself [32]. It is expected that interventions would have little to no effect without knowledge of driving factors such as the disease dynamics and the climate behind the cycle [33]. To some extent, knowledge of malaria prevalence trends could be useful in planning, monitoring and evaluating the level of implementation, or of interventions within a given treatment policy over time, such as the one ongoing in Cameroon. This could detect early warning signs of drug resistance development, such as treatment failure or a sustained upsurge in the number of malaria cases, despite receiving appropriate treatment. The objectives of this study were, therefore, to identify possible trends in malaria prevalence over time and in terms of season; to assess differences in malaria prevalence across gender and age groups and to evaluate the impact of changes to treatment guidelines on malaria prevalence. Furthermore, malaria drug resistance is known to develop within an average of 10–15 years [34], and thus, seven years following the introduction of ACT could be good time to investigate the trends.

The study is designed to understand the trends in malaria admissions over the period 2006–2012 following the change from chloroquine to ACT as first-line treatment and the effect of supplementary interventions in this area. The study site is Mbakong, a rural area located in lower Bafut along the Mezam and Menchum Rivers near the Menchum fall that has been earmarked for the generation of hydro-electricity. An in-depth understanding of the malaria prevalence in patients seeking treatment in this area will not only be of importance to the local population, but for government to improve its planning strategy for scaling up of malaria control programmes in rural areas and the affluent population that would be working on the dam or tourists visiting the Menchum fall. It will provide baseline data for the prospective studies on malaria prevalence in Cameroon.

## Methods

### Study site and population

**Figure 1:**
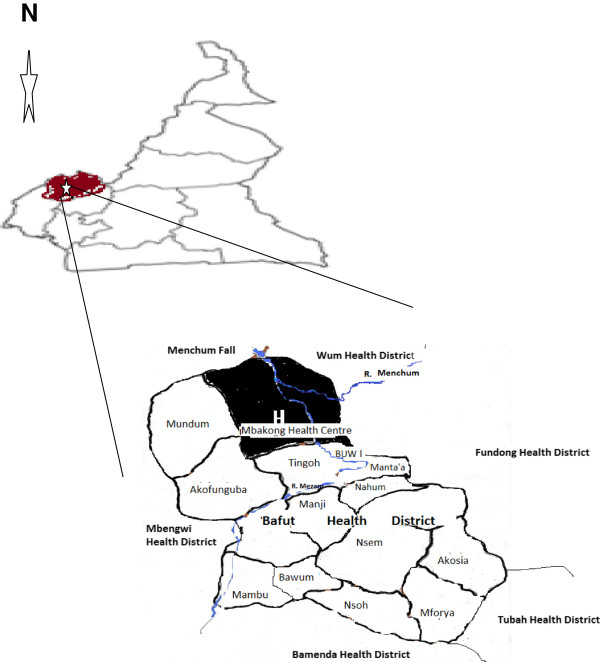
**The map of Cameroon showing the location of the study site.**

The Mbakong Health Area consists of riverine communities with GPS coordinates 6°10’18.87” N and 10°05.40” E with a head count of about 8,000 people. Mbakong falls within the Bafut Heath District of the North West Region of Cameroon (Figure 1). It lies within the Guinea Savannah region at the border of the Bafut forest on the South and the Bu forest to the north. It experiences a maximum rainfall pattern in August with a mean annual rainfall between 0 and 390 mm and a monthly temperature range of 22.49-26.34°C. Mbakong has two distinct seasons – the dry and the rainy season. The dry season starts from November to March while the rainy season starts from March through November. Human activities in this area include farming (maize and rice), game hunting and sand mining from the rivers. Sand mining is mainly carried out by young men (above 15 years of age). The women and children are involved in maize farming while all other members of the community are involved in rice farming.

### Study sample & data collection

Data was collected retrospectively through consultation and perusal of laboratory and prescription registers of the Mbakong Health Centre. Information was gathered on diagnosis, age, gender and the time (months and years) using operating procedures established for this study. The data was classified into six different age groups: <1 year (0–11 months), 1 to younger than five years (1 to <5), 5 to younger than 15 years (5 to <15), 15 to younger than 45 years (15 to <45), 45 to younger than 65 years (45 to <65) and 65 years and older (≥65). In this health facility microscopy was the only tool used for diagnosis. There was unfortunately no data on the environmental factors covering the seven years considered in this study and thus there is no attempt to assess the impact of environmental factors such as rainfall or temperature on the prevalence.

### Data analysis

Data analysis was conducted using SPSS Statistics V16.0 and SAS [35, 36]. All clinical diagnoses for which laboratory confirmations were requested but the results were not obtained were treated as missing data. Given that the sample was not randomly selected and cannot be established as representative of a larger population, the analyses focused on the practical significance of findings even though statistical significance is still reported [37]. Curve estimation was used to evaluate the significance of trends in the data. Pearson’s Chi Square test for relationship among variables was used to evaluate the malaria prevalence across gender and age groups. Finally, proportions and odds ratios were evaluated with regards to statistical and practical significance respectively.

### Ethical issues

The ethical clearance for this work was obtained from the Ministry of Public Health and the Institutional Review Board of the Catholic University of Cameroon, Bamenda. In order to maintain anonymity, all patients were given codes during the data extraction process and patient names were not transcribed from the register.

## Results

### The impact of changes to treatment guidelines on malaria prevalence among febrile patients seeking treatment

**Figure 2:**
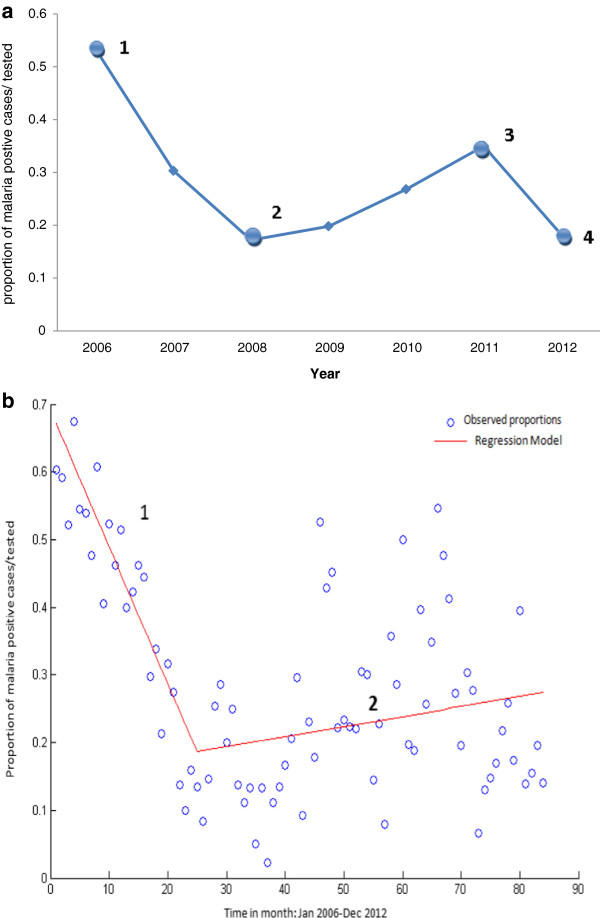
**Distribution of malaria positive cases over seven years at the Mbakong Health Centre 2006–2012. a)** Numbered dots indicate when different interventions were introduced: 1) implemented change in malaria treatment policy from monotherapy to ACTs, 2) New treatment guidelines made available to healthcare workers, 3) Nationwide distribution of 8 million LLIN and re-introduction of community management of malaria, 4) Roll out of RDTs for testing before treatment both at the community and health facility level. **b)** Piecewise Regression of admissions over months illustrating the change in prevalence from January 2006 to December 2012: Line 1 represents a sharp decline in malaria prevalence from 2006–2008 and Line 2 represents a gradual rise in prevalence from 2008–2012.

A total of 4,230 febrile patients sought treatment at the Mbakong Integrated Health Centre and were clinically diagnosed to be suffering from malaria from 2006–2012. Microscopy was conducted for 4,158 (98.30%) patients to confirm the presence or absence of parasites. Of these, 1,239 (29.30%) were confirmed positives with 15 patients per month on average. However, this number fluctuates dramatically from month to month. Between 2006 and 2012 confirmed malaria positive cases (taken here as a percentage of those tested) fluctuated, dropping steadily from 356 (53.21%, 95% CI 0.49-0.57) in 2006 to 93 (17.20%, 95% CI 0.14-0.20) in 2008 and then gradually rose to 244 (35.00%, 95% CI 0.31-0.39) in 2011 and finally dropped to 91 (18.20%, 95% CI 0.15-0.22) in 2012 (Figure 2a).

Piecewise regression was used to identify any significant change in prevalence when observed over the 84 months spanning January 2006 to December 2012. Figure 2b indicates an inflection point at January, 2008, corresponding to the publication of new treatment guidelines. The first regression line (Figure 2b line 1) was replicated using linear model via curve estimation and had an R^2^ value of 0.827, indicating a practically significant decline (gradient was also statistically significant p < 0.001) in prevalence (overall statistical significance p < 0.001) from 2006–2008. The second regression line (Figure 2b line 2) was modelled using the same methods. However, none of the models were practically significant. These findings suggest that the implementation of the new treatment guidelines coincide with the decay of a declining trend in malaria prevalence.

### Trends in malaria prevalence among febrile patients over time with reference to seasonal variation

**Figure 3:**
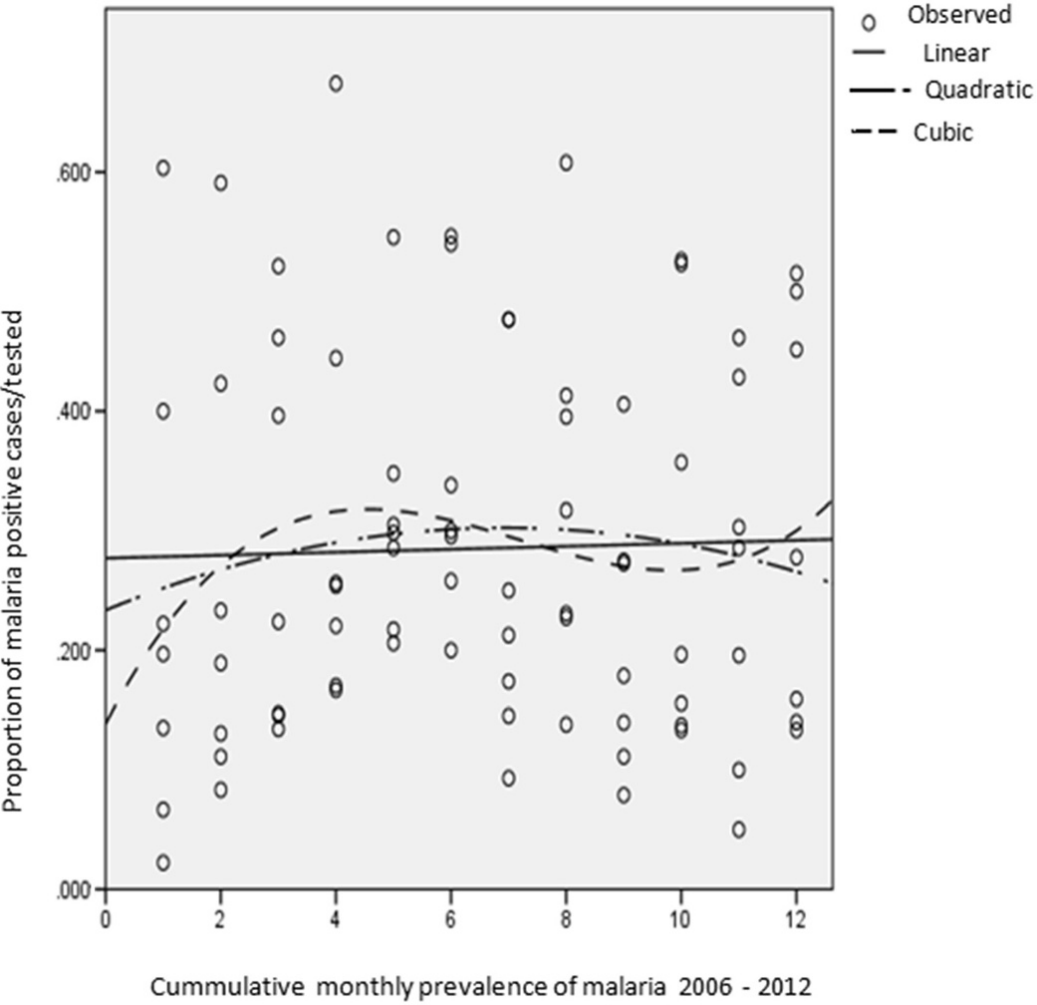
**Three models were used to investigate seasonal trend in malaria occurrence.** The graph shows monthly proportions fitted into the curve estimation model. Both confirm the absence of a seasonal trend as none of the models were statistically or practically significant.

The objective was to identify a possible seasonal trend over a 12-month period. The data used was the proportion of malaria positive cases of those tested observed for each month over the seven-year period. As described above, curve estimation was used to evaluate trend in the data. Three models were applied to the data to evaluated trends in the data over time, namely linear, quadratic and the cubic models. While there are a multitude of models that can be used to assess trends in the data over time, those selected here were deemed the most informative. The R^2^-values (representing the percentage variance explained by each model) were compared to identify the more appropriate model as well as to assess the goodness-of-fit of all three models. The R^2^-values were also used to assess the practical significance of trend with values above 0.5 indicative of a practically visible trend and values above 0.8 indicative of a practically significant trend [37]. Significance testing, at the 5% confidence level, was also performed to assess the overall significance of each model as well as the model coefficients. Figure 3 illustrates the absence of a seasonal trend as none of the models were statistically or practically significant.

To conclude, no seasonal trend could be established in the data. This is contrary to popular perception that there is a major transmission season during the rainy season between May and August and minor seasons between February to April and September to November. In addition, the perception of a December-January spike, in the heart of the dry season could be of interest since the mosquito population at this time is very low and consequently low malaria transmission. However, these perceptions could not be verified in the data used for this study. There is need to investigate further malaria prevalence in asymptomatic individuals from December through January in this area.

**Figure 4:**
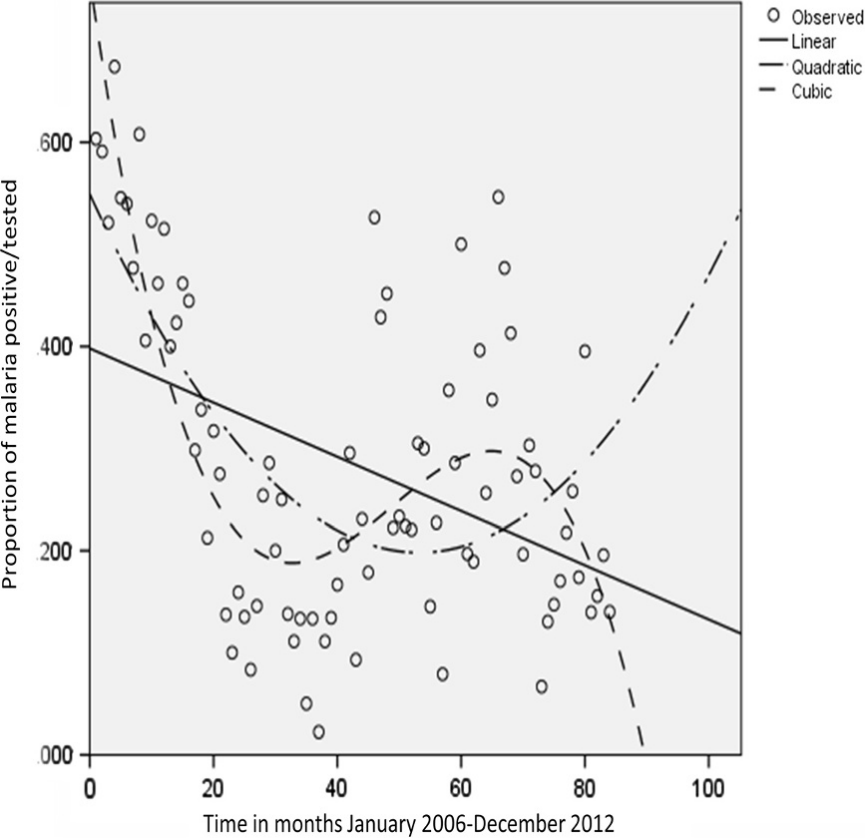
**Graph illustrating the distribution of the proportion of positive cases according to the months of the year over time using the curve estimation model (** **Linear Quadratic**, **and Cubic).** The cubic model was found to best fit the data with R^2^ = 0.638. Time is divided into 84 monthly observations and includes Jan. 2006 to Dec. 2012.

**Figure 5:**
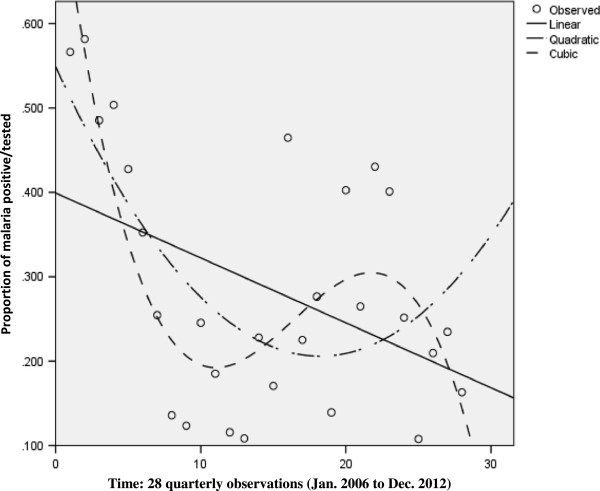
**Graph illustrating the distribution of positive cases according to the quarters of the year over time using the curve estimation model:** **Linear Quadratic**, **and Cubic?** The cubic model was found to best fit the data with R^2^ = 0.638.

Curve estimation was again employed to identify predefined trends over time. The data used was the proportion of malaria positive cases to those tested observed for each month over the seven-year period; 84 observations in total. Figure 4 illustrates the three models fitted to the prevalence over time. All three models were statistically significant; however, only the cubic model explained sufficient variation to be of practical relevance (R2 = 0.571, p<0.0001). The linear parameter of the cubic model was both negative and significantly different from zero (β=-0.042, p<0.0001). Proportions were also calculated over the 28 quarters (each year is divided up into four quarters of three months each) for the seven year period. This was done to reduce some of the variation in the observed monthly data in an attempt to identify trends. A cubic trend was found to be statistically significant and practically visible (R2 = 0.638, p<0.0001) (Figure 5). The coefficient for the linear component for the cubic model was significant as well as negative (β= -0.132, p<0.001). Both the R2, as well as the coefficient values were greater for the prevalence modelled per quarter, which further established a fluctuating yet declining trend.

### Malaria prevalence among febrile patients across gender and age groups

The distribution of confirmed malaria cases as a percentage of those tested was evaluated across gender and age groups for the period 2006–2012. Malaria was found to have a similar prevalence in females with 798 cases of those tested (29.20%, 95% CI 0.28-0.31) and males with 441 of those tested (30.90%, 95% CI 0.29-0.33) across all age groups. The males had a slightly higher risk of contracting malaria than the females (OR = 1.08, 95% CI 0.94-1.25, p = 0.26) though it was not practically significant.

The number of malaria confirmed cases of those tested at the Mbakong health centre varied across different age groups. In those tested, the age groups hardest hit by malaria were the five to <15 years (296; 40.00%, 95% CI 0.36-0.44), 1 to <5 years (373; 38.26%, 95% CI 0.35-0.41) and 15 to <45 years (411; 25.23%, 95% CI 0.23-0.27). Prevalence was found to be low in the age groups younger than 1 year old children, 45 to <65 years and 65 years or older patients with 400 (19.25%, 95% CI 0.15-0.23), 60 (19.481%, 95% CI 0.29-0.33) and 106 (20.76%, 95% CI 0.13-0.28), respectively. Overall, among the 1,239 patients who tested positive for malaria, children younger than five years of age constituted 36.30% while those younger than 15 years of age constituted 60.20% in Mbakong Health Centre.

The distribution of malaria prevalence among febrile patients across age was evaluated within each gender. Evaluating whether the observed differences in age within each gender are significant required grouping of the ages into categories, similar to those used in categorizing treatment regimes. Below 15 years of age, malaria prevalence was found to be similar between the male and the female children with the respective proportions 35.4% and 35.14%, except for patients younger than one year where the malaria admissions were found to be more in males (23.76%) than females (14.64%). Above the age of 15 years malaria prevalence was higher in the females (25.23%) than the males (19.4%), except among the ≥65 year old patients where it was similar. The odds of having a positive result for malaria was slightly higher in the males than in the females for patients younger than one year (OR = 1.82, 95% CI 1.09-3.03 p = 0.02), 5 to <15 years (OR = 1.03, 95% CI 0.77-1.39, p = 0.833) and ≥65 years (OR = 1.07, 95% CI 0.41-2.78, p = 0.98). It is worthy of note that odds ratios were not statistically significant except for patients younger than one and none were of practical relevance [37]. In the age group 1 to <5 years and 15 to <45 years, the risk of having malaria was higher in the females compared to the males (OR = 1.14, 95% CI 0.88-1.47, p = 0.33) and (OR = 1.29, 95% CI 0.95-1.75, p = 0.097), respectively, without any practical significance. The females in the age group 45 to <65 years had a higher risk of having malaria than the males, which was both statistically significant and practically visible (OR = 2.87, 95% CI 1.18-7.01, p = 0.016).

The data revealed that from 2006 to 2008 malaria prevalence among febrile patients decreased in all the age groups. A drastic drop was witnessed in the following age groups in the females: <1 year (from 54.55% in 2006 to 9.09% in 2008 and then to 0% in 2009) and 1 to <5 years (75.56% in 2006 to 15.25% in 2008), while in the males it dropped drastically in the following age groups: <1 year (50.00% in 2006 to 15.25% in 2008), 1 to <5 year old (60.87% in 2006 to 9.84% in 2008) and finally from 40 and 50% in 2006 respectively, in the age group 45 to <65 and ≥ 65 years group to 0% in 2008. Pearson’s product–moment correlation coefficient was calculated to test the association between prevalence and the ages (ungrouped) of patients. The association was found to be both statistically as well as practically significant (r = -0.55; p < 0.001) with lower levels of prevalence observed in older patients.

**Figure 6:**
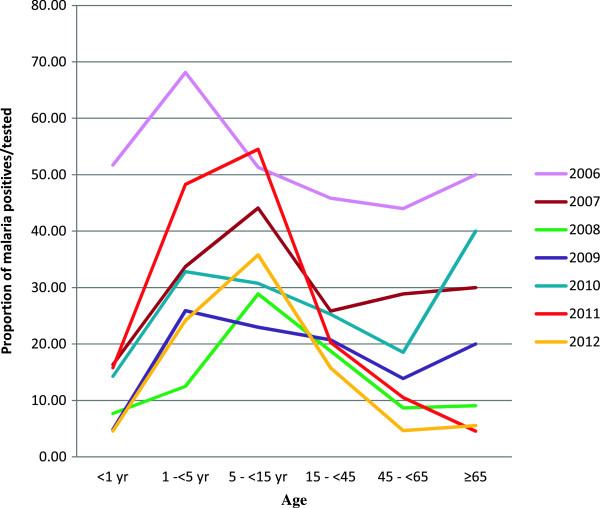
**Graphs depicting malaria prevalence as a proportion of those tested over the study period across different age groups from 2006–2012.** The age group 1 to under 5 years and 5 to under 15 years old children are revealed to be the most affected across all the years and were the hardest hit during the upsurge in 2011.

In 2008, malaria prevalence in febrile patients seeking treatment was relatively higher in the females than males while males maintained relatively higher prevalence from 2009 through 2012. From 2008 through 2011, the age group 1 to <5 years and 5 to <14 year old children for both genders observed an increase in prevalence. In males the increase was from 9.84% in 2008 to 51.28% in 2011 in the 1 to <5 year olds while in the 5 to <15 year olds it increased from 20.56% in 2008 to 59.77% in 2011. In the females the age group 1 to < 5 year old witnessed a similar rise from 15.25% in 2008 to 45.83% in 2011 while the 5 to <15 year old group witnessed a fluctuating rise from 35.85% in 2008 to 50.00% in 2011. Following the 2008 drop in malaria, the age group 15 to <45 year old has maintained a fluctuating but stable prevalence over the last five years 2008–2012. From the analysis the age groups 1 to <5 and 5 to <15 year old children seem to have been the major affected age groups during the upsurge in malaria prevalence witnessed in 2011 (Figure 6).

Pearson’s Chi Square test for degree of association was used to investigate significant differences in prevalence between years, months of each year, age groups and gender (based on the number of malaria positive cases). The test revealed a practically visible association between age and gender (Pearson ×^2^ = 153.675, p < 0.001, Cramer’s V = 0.352). A very weak association between year and age (Pearson ×^2^ = 156.413, p < 0.001, Cramer’s V = 0.159) was observed. No significant association was found between month of the year and age (Pearson ×^2^ = 68.469, p = 0.105, Cramer’s V = 0.105). A very weak association was found between year and gender (Pearson ×^2^ = 13.085, p = 0.042, Cramer’s V = 0.105) and no association was found between gender and month of the year (Pearson ×^2^ = 5.510, p = 0.94, Cramer’s = 0.067).

An analysis of the data revealed that 40/4230 (1.05%) febrile patients returned for treatment within one month of treatment for malaria at the Mbakong Health Centre with 23 (57.5%) being males while 17 were females. Of these 15 (37.5%) were <1 year infants (13 males and 2 females), 12 (30%) 15–49 years, 6 (15%) 15–14 years, 5(12.5%) 1–4 years and 2 (5%) were 50–64 years. Twenty-six (65%) of the returning patients were children ≤15 years. The number of returning patients following treatment at Mbakong witness a dropped from 11 (27.5%) in 2006 to three (7.5%) in 2010 and 2011 and four (10%) in 2012. Of all the returning cases only 17 (42.5%) tested positive for malaria (nine females and eight males). Interestingly the number of returning cases increased tremendously to 28 patients in 2013 (Additional file 1).

## Discussion

Microscopy was well utilized at the Mbakong Health Centre with 98.30% of cases tested for malaria. This demonstrates an effective use of the testing tool at the level of this health centre. This observation contrasts with earlier reports that observed that microscopy was widely available in public and private facilities, but underused, with less than half of patients tested for malaria during their consultation [5, 11]. However, this conclusion is drawn from an observation in one public facility and could deviate from this value as more facilities and different regions are included.

The results of the present study have revealed a malaria prevalence of 29.80% for tested febrile patients seeking treatment within the study period 2006–2012 in the Mbakong Health Area. The result tallied with an earlier report of a clinical trial by Mangham and colleagues [5], who observed that 29% of febrile outpatients that visit either a public or private health facility or medicine retail store were positive for malaria in Bamenda and Yaoundé. This was further supported by a report that asymptomatic pregnant women attending the Antenatal clinic in the Bafut Health District revealed a malaria prevalence of 28.4% (Engochan Mbakwa, Ignatius Ndong, Wilfred Mbacham and colleagues, 2014, personal communication). The malaria prevalence among febrile patients seeking treatment in Mbakong is lower than the 37% reported for rural areas and higher than the 15% reported for the North West region of Cameroon [20]. However, the malaria prevalence in febrile patients observed in the study was 10% lower than the 39.6% of confirmed malaria cases reported in a similar study in Ethiopia [30]. The fluctuating patterns from 2006–2012 observed in this study were very similar to those reported in studies in Ethiopia and Rwanda [30, 38] with a one-year lag period. While the lowest and peak values in our study were observed in 2008 and 2011, respectively five years after the 2006 surge, those in the Ethiopian study were observed in 2007 and 2010 respectively [30]. It is not clear what the influence of factors such as climate and environment in this regard are across the continent on the malaria cycle.

This observation closely ties in with five-year resurgence cycle of malaria. No seasonal trends were observed. Over time (taken as observations in month as well as quarters) there was a significant and practically relevant fluctuating but progressive decline in prevalence.

Since there was a significant association between the gender, age groups and malaria positives cases, future research will be required to investigate the possibility of using age group and gender to predict the future outcome of a patient tested for malaria in Mbakong and surrounding areas. However, substantial improvement seems to have been achieved with the various interventions from 2006–2012. This can be seen from the fact that annual malaria prevalence in febrile patients in 2011 was about 18% less than that of 2006 and though fluctuating, malaria admissions appear to be on the decline at the Mbakong Health centre [17].

The drop in malaria related admissions witnessed in 2012 coincides with the scaling up of the nationwide free distribution of long lasting insecticide treated nets and the roll out of RDT for community relay agents to conduct a confirmation test before treating for malaria by the National Malaria Control Programme [23, 24, 39]. If the latter had an effect, it seems to have been short-lived as the prevalence gradually rose in the course of 2012. This may point to the fact that the malaria prevalence in the area follows its natural cycle despite the drug and insecticide pressure [32] or that the population were excited and used the nets in the first few months and then relaxed. Alternatively, there may be a growing resistance to the insecticides used on the nets [23, 24, 40–42]. However, the impact of different interventions under the new policy on the level of malaria, though not yet fully investigated, cannot be under estimated. This can be seen from the fact that prevalence level within the study period even in the upsurge phase in 2011 never attained the 2006 levels. One of the setbacks in this study is the lack of data before 2006 at the health centre, making comparison to pre-2006 prevalence impossible. However, the 2006 upsurge in prevalence observed in this study coincides with those reported in a similar study in Ethiopia and Rwanda [30, 38] and seems to closely match the periods between 2000–2010 during which upsurge in malaria mortality was observed in the systemic review by Murray et al*.*[4].

Malaria prevalence in the febrile children under five years of age is higher than those reported by Oladosu & Oyibo [43] and Alemu et al. [30], but consistent with similar findings in South Western Cameroon where higher parasitaemia in asymptomatic children has been reported [44]. Furthermore, the fact that children below age 15 years constitute 60.2% of the total malaria positive cases detected in this health centre is in line with earlier reports that children bear the greatest burden of malaria in the world [45]. No clear pattern was found in terms of gender and age groups but correlation tests shows that as age increases, malaria prevalence tends to decrease, corroborating an earlier finding in Tanzania that malaria prevalence decreases with age [46]. This could be a result of the partially acquired immunity cumulated with age and repeated infections with the *Plasmodium* parasites [47]. While a declining trend in prevalence was witnessed by all age groups from 2006–2008, almost all the groups witnessed an upward trend from 2009–2011 except the age group 15 to <45 years, which maintained a fluctuating trend from 2009–2012. Data analyses reveal that the upsurge in malaria prevalence observed in 2011 was due to a drastic increase in prevalence in two age groups, 1 to <5 year and 5 to <15 year old children. The exact cause of this is unclear. These age groups fall within those with low partial immunity in an endemic area. The findings in this study suggest that, amongst those tested, the most affected groups are children and women. This result is in sharp contrast with the report by Alemu et al*.*[30] where men where the most affected. Although farming is the common denominator in the most affected groups in both studies, it is not yet clear how this contributes to the high prevalence in this young age group.

Though the odds of having positive results for malaria alternated between males and females from one age group to the other the effect size was small. Nevertheless, the chances of having malaria were found to be generally higher in the males than the females. However, females in the age group 45 to <65 years had a significantly higher risk of a positive malaria result. This was supported by a practically visible effect (p = 0.026 OR = 2.87234).

Though there was dropped in the number of returning cases treated for malaria at the Mbakong Health Centre from 2006–2012 which could be attributed to various interventions under the present policy. An interesting picture was observed in the high number of return cases in 2013, which was beyond this study period. Though 42.5% of the returning febrile patients tested positive for malaria, there is, however, no clue than this return rate of 1.05% was due to treatment failure using ACT since there was no patient follow up in this study. This could be a pointer that there is need to monitor for treatment failure at this health centre. This study acts as a baseline for future studies on the efficacy of ACT and other interventions.

### Limitation of the study

The findings of this study are limited to the Mbkong health centre and based on clinical and microscopic diagnoses records of febrile patients considered retrospectively over a seven-year period 2006–2012. Prevalence of malaria earlier than 2006 is not known as records before this period were not available. Data on asymptomatic malaria in adults is not captured in this study as it focuses on hospital records of essentially febrile patients seeking treatment.

## Conclusions

The findings of this study reveal that the malaria admissions in Mbakong have shown a fluctuating, but declining trend over the last seven years with a practically relevant significance. The Mbakong Health Centre seems to have witnessed a significant decline in the malaria admissions in the different age groups. Though it is not clear whether the observed decline was due to the intervention under the current policy or part of the natural cycle of malaria, the impact of the new treatment policy cannot be under estimated. There is need for control efforts to be stepped up and for establishing a monitoring scheme with specific reference to the vulnerable groups.

One of the cyclic phases of the malaria disease with a declining trend seems to have been witnessed within this seven year period of 2006–2012. However, the study period needs to be extended and the number of facilities increased for meaningful conclusions to be drawn. Nevertheless, this study provides a baseline for malaria prevalence studies to be conducted in this area in future.

## Electronic supplementary material

Additional file 1: **Table depicting the number of febrile patients who returned to the Mbakong health centre within one month of an initial malaria treatment.** (DOCX 15 KB)
