# Supplementary material for: Trends in malaria admissions at the Mbakong Health Centre of the North West Region of Cameroon: a retrospective study
Source: Malar J. 2014 Aug 22;13:328. doi: 10.1186/1475-2875-13-328 (PMC4247771; doi:10.1186/1475-2875-13-328)
Supplement: Supplementary file 1 — Additional file 1: Table depicting the number of febrile patients who returned to the Mbakong health centre within one month of an initial malaria treatment. (DOCX 15 KB) [file 12936_2014_3602_MOESM1_ESM.docx]

Table depicting the number of febrile patients who returned to the Mbakong health centre within one month of an initial malaria treatment.

|  | **0-11Month**  **n=15** | | **1-4 years**  **n=5** | | **5-14 years**  **n=6** | | **15-49year**  **n=12** | | **50-64 years**  **n=2** | | **≥65 years**  **n=0** | | **Total per gender**  **n=40** | | **Annual total** |
| --- | --- | --- | --- | --- | --- | --- | --- | --- | --- | --- | --- | --- | --- | --- | --- |
|  | **Male** | **Female** | **Male** | **Female** | **Male** | **Female** | **Male** | **Female** | **Male** | **Female** | **Male** | **Female** | **Male** | **Female** |  |
| 2006 | 4 | 1* | 0 | 1* | 2/1* | 1 | 0 | 2 | 0 | 0 | 0 | 0 | 6/1* | 5/2* | 11 |
| 2007 | 4/2* | 0 | 0 | 1* | 0 | 0 | 0 | 1 | 1 | 0 | 0 | 0 | 5/2* | 2/1* | 7 |
| 2008 | 4/3* | 0 | 0 | 1* | 0 | 0 | 0 | 1* | 1 | 0 | 0 | 0 | 5/3* | 2* | 7 |
| 2009 | 0 | 0 | 1* | 0 | 2 | 0 | 0 | 2/1* | 0 | 0 | 0 | 0 | 3/1* | 2/1* | 5 |
| 2010 | 1 | 0 | 0 | 1* | 0 | 0 | 1* | 0 | 0 | 0 | 0 | 0 | 2/1* | 1* | 3 |
| 2011 | 0 | 0 | 0 | 0 | 0 | 1 | 0 | 2 | 0 | 0 | 0 | 0 | 0 | 3 | 3 |
| 2012 | 0 | 1* | 0 | 0 | 0 | 0 | 2 | 1* | 0 | 0 | 0 | 0 | 2 | 2* | 4 |
| **Total** | **13/5*** | **2*** | **1*** | **4*** | **4/1*** | **2** | **3/1*** | **9/3*** | **2** | **0** | **0** | **0** | **23/8*** | **17/9*** | **40/17*** |
|  |  |  |  |  |  |  |  |  |  |  |  |  |  |  |  |
| 2013 | 1* | 1* | 1 | 3/1* | 1 | 4 | 4 | 2 | 2 | 2 | 4 | 3 | 13 | 15 | 28 |
| **Total** | **14** | **3** | **2** | **7** | **5** | **6** | **7** | **11** | **4** | **2** | **4** | **3** | **36** | **32** | **68** |

*refers to number returning febrile patients confirmed positive for malaria.

An analysis of the data revealed that 40/4230 (1.05%) febrile patients returned for treatment within one month of treatment for malaria at the Mbakong health centre with 23 (57.5%) being males while 17 were females. Twenty-six (65%) of the returning patients were children ≤15 years. Of these 15(37.5%) were ˂1 year infants (13 males and 2 females), 12(30%) 15-49 years, 6 (15%) 15-14 years, 5(12.5%) 1-4 years and 2 (5%) were 50-64 years. The number of returning patients following treatment at Mbakong witness a dropped from 11(27.5%) in 2006 to 3(7.5%) in 2010 and 2011 and 4(10%) in 2012. Of all the returning cases only 17 (42.5%) tested positive for malaria (9 females and 8 males). Interestingly the number of returning cases increased tremendously to 28 patients in 2013. There was however no clue than this return rate was due to treatment failure since there was no patient follow up in this study. This study acts as a baseline for future studies on the clinical trials and efficacy of ACTs.
